# Comprehensive analysis of immune subtype characterization on identification of potential cells and drugs to predict response to immune checkpoint inhibitors for hepatocellular carcinoma

**DOI:** 10.1016/j.gendis.2024.101471

**Published:** 2024-11-27

**Authors:** Guichuan Lai, Biao Xie, Cong Zhang, Xiaoni Zhong, Jielian Deng, Kangjie Li, Hui Liu, Yuan Zhang, Anbin Liu, Yi Liu, Jie Fan, Tianyi Zhou, Wei Wang, Ailong Huang

**Affiliations:** a Department of Health Statistics, School of Public Health, Chongqing Medical University, Chongqing 401331, China; b Department of Applied Statistics, School of Public Health, Chongqing Medical University, Chongqing 401331, China; c Department of Epidemiology, School of Public Health, Chongqing Medical University, Chongqing 401331, China; d Key Laboratory of Molecular Biology for Infectious Diseases (Ministry of Education), Institute for Viral Hepatitis, Department of Infectious Diseases, The Second Affiliated Hospital, Chongqing Medical University, Chongqing 400010, China

**Keywords:** Cellular heterogeneity, Drug prediction, Hepatocellular carcinoma, Immune checkpoint inhibitors, Immunosubtyping

## Abstract

Immunosubtyping enables the segregation of immune responders from non-responders. However, numerous studies failed to focus on the integration of cellular heterogeneity and immunophenotyping in the prediction of hepatocellular carcinoma (HCC) patients' response to immune checkpoint inhibitors (ICIs). We categorized HCC patients into various immune subtypes based on feature scores linked to ICI response. Single-cell sequencing technology was to investigate the cellular heterogeneity of different immune subtypes and acquire significant ICI response-associated cells. Candidate drugs were identified using a blend of various drug databases and network approaches. HCC patients were divided into two distinct immune subtypes based on characterization scores of 151 immune-related gene sets. Patients in both subtypes showed varying overall survival, immunity levels, biological activities, and TP53 mutation rates. Subtype 1-related natural killer cells showed a positive correlation with immune-promoting scores but a negative correlation with immune-suppressing scores. Notably, docetaxel sensitivity in HCC patients rose as the levels of subtype 1-related natural killer cells increased. Our study demonstrated that immune subtypes have cellular heterogeneity in predicting response to ICIs. A combination of subtype 1-associated natural killer cells and docetaxel may offer new hope for ICI treatment in HCC.

## Introduction

Globally, liver cancer is the third most common cause of cancer-related deaths.^1^ It is estimated that there will be 1.4 million new diagnoses and 1.3 million deaths by 2040, with hepatocellular carcinoma (HCC) accounting for 80 % of the overall liver cancer incidence.^1^^,^^2^ The primary approaches for managing HCC include hepatectomy, radiotherapy, and hepatic artery perfusion chemotherapy.^3^ Furthermore, there is a range of new medications accessible for first- and second-line treatment, especially immune checkpoint inhibitors (ICIs), which are now the first-line treatment for patients with advanced HCC.^4^^,^^5^ However, despite ICIs yielding a superior therapeutic impact in HCC, a substantial number of patients remain resistant to this breakthrough therapy.^6^ Current challenges in the use of ICIs include the absence of biomarkers for predicting the response to ICIs and the reliability of existing markers that still need to be validated.^7^^,^^8^ For instance, a preliminary study on nivolumab in patients with HCC discovered that the tumor cell programmed cell death-ligand 1 status at baseline did not significantly affect the immune response rate and is yet to be recognized as a consistently dependable biomarker.^7^ Additionally, established markers for ICIs such as tumor mutation burden and microsatellite have not demonstrated significance in HCC.^8^ Hence, it is imperative to investigate fresh dependable biomarkers to enhance patient prognosis whilst lessening the adverse repercussions and economic strains of ICIs. ^9^

The identification of immune subtypes is essential in recognizing patients who have cold and hot responses to ICIs.^10^ Zhang et al discovered that HCC patients show varied degrees of tumor cell metabolism and cytokine expression, as well as prognostic differences, under various classifications based on immune cells. ^11^ ImmuneSigDB is a curated gene set designed for the analysis of transcriptome data concerning various cellular conditions, experimental manipulations, and genetic perturbations.^12^ Gong et al utilized ImmuneSigDB to classify HCC and predict patient prognosis^13^; however, our study conducts a more meticulous analysis of ImmuneSigDB focus on predicting HCC patients' response to ICIs. The immune cell distribution and heterogeneity have significant implications in determining suitable patients for ICIs.^14^ A single-cell sequencing study recently analyzed the immune microenvironment of HCC, revealing that tumor-associated neutrophils accumulate in the myeloid-rich subtype class, indicating their roles in immunosuppression.^15^ This indicates that the classification of immune cells could clarify the contribution of cellular diversity to ICIs. Hence, incorporating gene expression and immune cell distribution among patients with diverse immune subtypes is crucial to identifying the specific cells and molecules that may impact ICIs.

**Figure 1:**
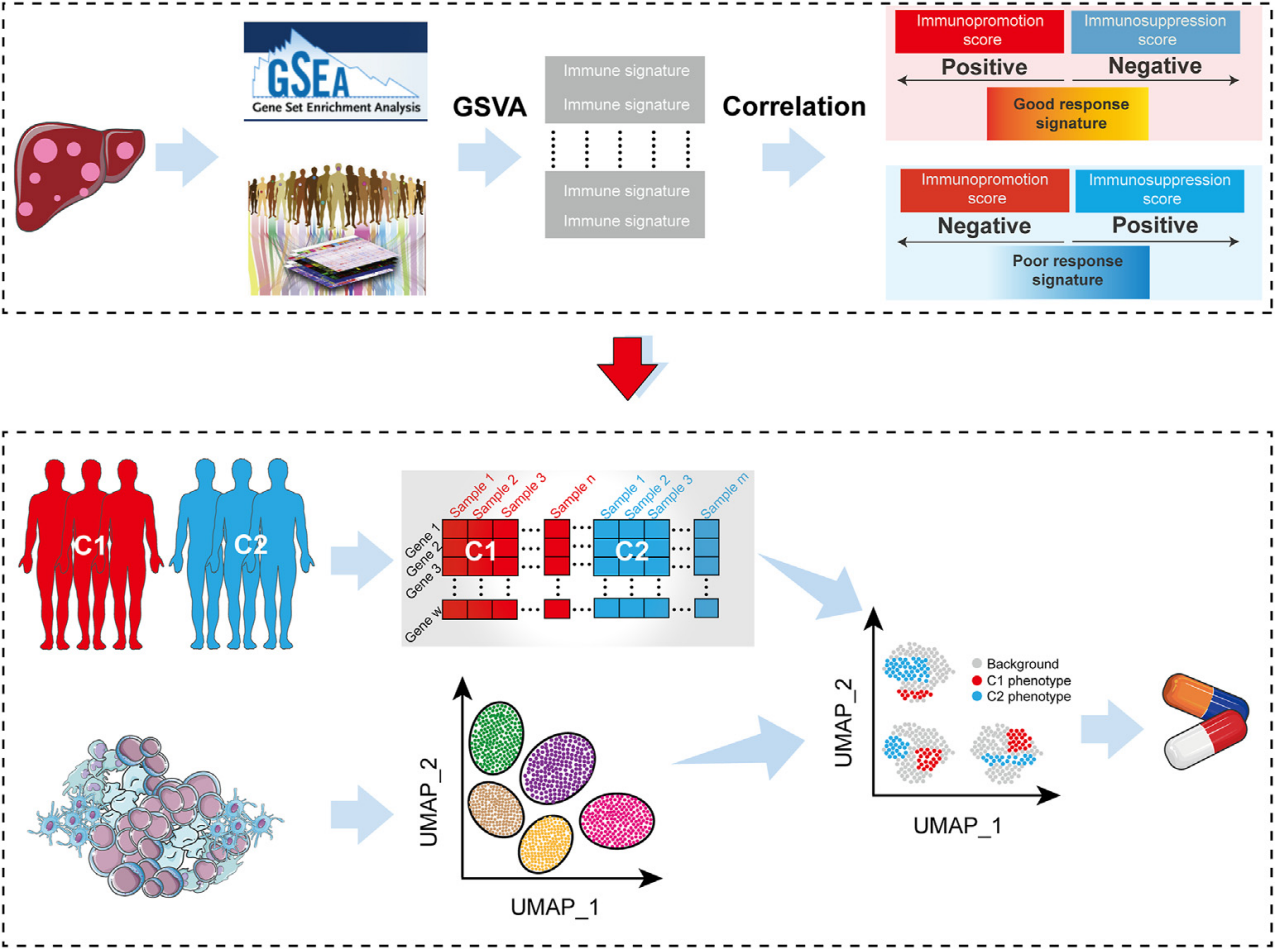
Schematic diagram of this study.

As shown in Fig. 1, we obtain the ImmuneSigDB signature scores of HCC patients and introduce seven algorithms that can predict the response to ICIs to obtain the gene sets associated with ICI response, and use unsupervised consensus clustering to identify the immune subtypes of HCC patients (bulk data of patients with different immune subtypes are effectively integrated with single-cell sequencing data to identify cells related to immune subtypes), and finally determine suitable candidate drugs. Our aim is to explore the heterogeneity of immune subtypes at the single-cell level using bulk and single-cell sequencing to identify potential ICI response-associated cells and therapeutic agents and to provide a scientific basis for the application of ICIs in HCC.

## Materials and methods

### Data download

High-throughput transcriptome sequencing data and clinical data from 365 HCC patients were downloaded from The Cancer Genome Atlas (TCGA) (http://xenabrowser.net/) database. The count values obtained were converted to transcripts per million values using the “immunedeconv” package,^16^ and the gene expression levels were estimated by log_2_(TPM + 1). Gene expression profiles and clinical data of 357 HCC patients were obtained from GSE14520 (https://www.ncbi.nlm.nih.gov/geo/query/acc.cgi?acc=GSE14520) and GSE76427 (https://www.ncbi.nlm.nih.gov/geo/query/acc.cgi?acc=GSE76427) datasets. Single-cell sequencing data from five HCC patients were obtained from GSE166635 (https://www.ncbi.nlm.nih.gov/geo/query/acc.cgi?acc=GSE166635) and GSE162616 (https://www.ncbi.nlm.nih.gov/geo/query/acc.cgi?acc=GSE162616) datasets. Besides, we obtained the IMvigor210 immunotherapy cohort data for patients who were treated with anti-PD-L1 inhibitors using the “IMvigor210CoreBiologies” package.^17^

### Identification of ICI response-related gene sets

We retrieved 4872 immune gene sets from the Molecular Signatures Database (MSigDB) (http://gsea-msigdb.org/gsea/index.jsp) and computed the respective immune signature scores for TCGA-HCC patients via the “Gene Set Variation Analysis (GSVA)” algorithm.^18^ To obtain gene sets connected with response to ICIs, we applied five classic ICI prediction algorithms, namely, “Tumor Immune Dysfunction and Exclusion (TIDE)”,^19^ “immunophenoscore (IPS)”,^20^ “MHC I association immunoscore (MIAS)”,^21^ “IMmuno-PREdictive Score (IMPRES)”,^22^ and “T cell-inflamed gene expression profile (GEP)”^23^ algorithms, and determined gene sets with response effects through the correlation between immune signature scores and these five scores. Additionally, we included algorithms that calculate immune microenvironmental scores, namely “ESTIMATE”^24^ and “xCell”.^25^ TIDE score displays a negative correlation with response to ICIs, and the other four scores (IPS, MIAS, IMPRES, and GEP) display a positive correlation with response to ICIs, it is worth considering that responders typically possess more immune-related traits than non-responders. Therefore, we specified that the GSVA score for the good response-associated gene set was positively correlated with the IPS, MIAS, IMPRES, GEP, ESTIMATE-immunescore, and xCell-immunescore, and negatively correlated with the TIDE score. Conversely, the GSVA score for the poor response-associated gene set was negatively correlated with IPS, MIAS, IMPRES, GEP ESTMATE-immunescore, and xCell-immunescore, and positively correlated with the TIDE score.

### Classification of immune subtypes

Based on the feature scores of the ICI response-related gene sets, we employed unsupervised consistent clustering utilizing K-means clustering and built-in Euclidean distances for the immune-related typing of TCGA-HCC patients through the “ConsensusClusterPlus” package.^26^ We determined the optimal number of clusters based on the minimum Proportion of Ambiguous Clustering values.^27^

### Characterization of ICI-related features

In this study, we compared the differences in three aspects related to ICIs, including somatic mutations, pathways, and tumor-infiltrating immune cells in patients with different immunophenotypes. Firstly, mutation data from TCGA-HCC patients were acquired using the “TCGAbiolinks”^28^ and “maftools”^29^ packages. Secondly, 50 hallmark pathways from the MSigDB database were obtained, and the GSVA algorithm was employed to determine the enrichment scores of TCGA-HCC patients on these pathways. In addition, we identified the pathways related to the immune subtypes using the gene set enrichment analysis (GSEA) algorithm. Finally, we used “CIBERSORT”,^30^ “CIBERSORT-ABS”, “OUANTISEQ”,^31^ and “xCell”^25^ algorithms to assess the immune microenvironmental profile of TCGA-HCC.

### Determination of cells associated with immune subtypes

The study subjects, which were analyzed using single-cell sequencing in the datasets GSE166635 and GSE162616, respectively, were from Shanghai Oriental Hepatobiliary Surgery Hospital and the First Affiliated Hospital of the University of Science and Technology of China. Written informed consent was obtained from all patients, as approved by the ethics committees of their hospitals. Firstly, we processed the single-cell data for quality control. We filtered the cells based on the criteria that they expressed at least 200 genes and no more than 3000 per cell, with the number of UMI detected per cell being less than 12,000 and the mitochondrial ratio being less than 0.1. Additionally, we filtered genes to include only those expressed at least 3 cells. The LogNormalize method included in the NormalizeData function was used to normalize the single-cell sequencing data. The batch-to-batch effect between different samples was effectively mitigated by the “Harmony” algorithm, the optimal number of components was determined based on the inflection plot, and the results of the single-cell clustering were visualized by the “uniform manifold approximation and projection (UMAP)” algorithm.^32^ In this study, on the basis of automated annotation finished by the “SingleR” algorithm,^33^ we obtained the marker genes from CellMarker2.0 (http://117.50.127.228/CellMarker/) database and used manual annotation to determine the final annotation results. The “CopyKat” algorithm is used to detect malignant cells from epithelial cells.^34^ The algorithm classifies cells with a prediction of “Aneuploid” as malignant and those with a prediction of “Diploid” as normal. We integrated the expression matrix of TCGA-HCC, immune subtypes, and annotated single-cell expression matrix, and used the “Scissor” algorithm to predict cells associated with immune subtypes based on the principle of binary variable regression.^35^ The principle of the “Scissor” algorithm can be found in Figure S1.

### Calculation of the proportion of cells associated with immune subtypes

The “hdWGCNA” algorithm is utilized for identifying the hub genes linked to cells at the single-cell level.^36^ The rationale for the algorithm can be found in Figure S2. The genes expressed in at least 5% of cells were retained, and metacells were constructed based on cell types. The “TestSoftPowers” method was utilized for calculating varied soft thresholds. When the scale-free topological model has a fitting value exceeding 0.8 initially, the optimal soft power threshold is reached. The module's eigenvalue provides an objective measure of its overall situation. We calculated each cell's corrected module eigenvalue. Hub genes are those highly connected within each module. We obtained hub genes based on their eigengene-based connectivity (kME) values using the “ModuleConnectivity” method. The “Seurat” algorithm was utilized to acquire the activity scores of the top 25 hub genes in each module. The key gene modules were identified based on the activity scores. This led to the final acquisition of the specific genes for the immune subtype-associated cells. In accordance with the kME value, the top 25 hub genes were identified as specific genes for immune subtype-associated cells. The “ssGSEA” algorithm was employed to calculate the proportion of immune subtype-associated cells.^18^

### Prediction of drug candidates

To enhance the accuracy of identifying ICI response-associated cells, we implemented correlation analysis by examining the proportion of immune subtype-linked cells and the seven ICI response-based predictive scores. Using established criteria for identifying immune gene sets related to ICI response, we were able to identify potential ICI response-associated cells. For genes specific to candidate ICI response-associated cells, we acquired drugs from the DRElMT (http://www.dreimt.org/query/drug-prioritization) database that may modulate these cells, and only utilized drugs that have already been approved. To confirm that drug targets possess therapeutic potential for HCC, we searched the GeneCards (http://genecards.org/) database for non-duplicated genes related to HCC using the search terms “hepatocellular carcinoma”, “hepatocellular cancer”, “liver hepatocellular carcinoma”, or “liver hepatocellular cancer”.^37^ A total of 12,500 HCC-associated non-duplicated genes were collected (Table S1). We then constructed a network of interactions linking the drug targets and disease genes. We secured the background files for the network from the String (http://cn.string-db.org/cgi/input?sessionId=bq3ONpHVnJ16&input_page_show_search=on) database. We applied the “Between”, “Closeness”, “Degree”, “EPC”, “MCC”, “MNC”, “Radiality”, and “Stress” algorithms with the cytoHubba plug-in incorporated in the Cytoscape3.7.1 software to identify the network's top 10 genes based on node importance and frequency of appearance in the above algorithms. These genes represent potential drug targets. To ensure the effectiveness of the drug against HCC, we scrutinized the key drug candidates available in the DrugBank (http://go.drugbank.com/) and DGIdb (http://dgidb.org/) databases. This stage ensures that the drug candidates we select possess both anti-tumor and immune effects and have been tested in ongoing or completed trials for HCC. To acquire the 2D structures of the drug candidates, we referred to the Pubchem (http://pubchem.ncbi.nlm.nih.gov/) database and then generated the 3D structures using ChemBio3D Ultra 14.0 software. The “pRRophetic” package was employed to predict the sensitivity of HCC patients to the drug candidates.^38^

### Statistical analysis

All analyses were conducted using R version 4.0.3. To evaluate the statistical significance of survival between groups (identified by varying immune subtypes and high/low levels of candidate cells), the Kaplan–Meier method was performed with log-rank tests. To test differences in expression levels of each group, the Wilcoxon method was used. Spearman's rank correlation analysis was utilized to investigate the correlation between two groups, namely immune signature scores and ICI-related predictive scores, candidate cell levels, and IC50 of candidate drugs. To evaluate differences in immune response rates and gene mutation rates among different immune subtypes, a Chi-squared test was employed. All test outcomes indicate statistically significant differences at a *P* value of less than 0.05.

## Results

### Tumor subtypes based on ICI response-associated gene set signatures have the potential to predict ICI response

**Figure 2:**
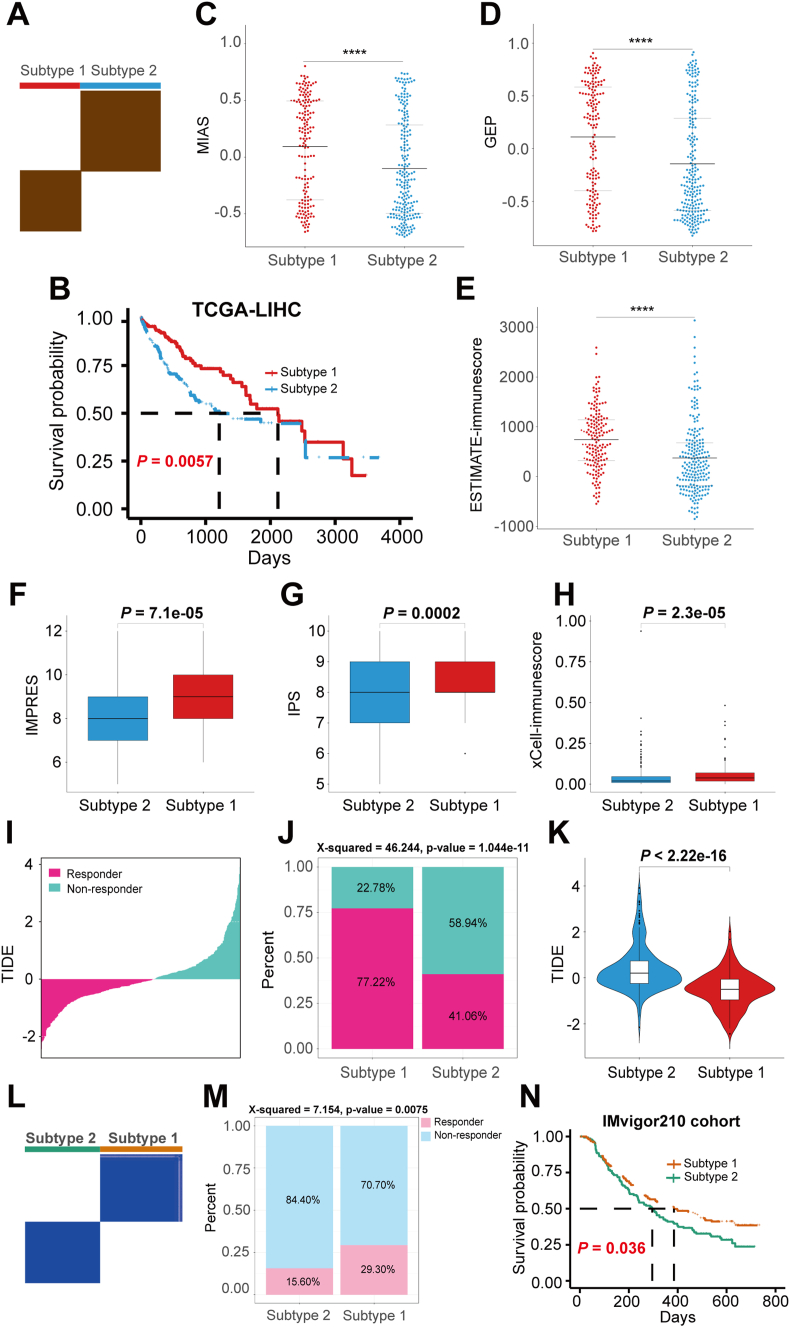
Identification of immune subtypes based on ICI response-related gene set feature scores. **(A)** Unsupervised consistent clustering of the two subtypes of TCGA-HCC patients. **(B)** Comparison of overall survival (OS) between the two subtypes of TCGA-HCC patients. **(C)** Comparison of MIAS scores between the two subtypes of TCGA-HCC patients. **(D)** Comparison of GEP scores between the two subtypes of TCGA-HCC patients. **(E)** Comparison of ESTIMATE-immunescore scores between two subtypes of TCGA-HCC patients. **(F)** Comparison of IMPRES scores between two subtypes of TCGA-HCC patients. **(G)** Comparison of IPS scores between two subtypes of TCGA-HCC patients. **(H)** Comparison of xCell-immunescore scores between two subtypes of TCGA-HCC patients. **(I)** Distribution of TIDE scores between responders and non-responders in TCGA-HCC patients predicted by the TIDE algorithm. **(J)** Comparison of response rates between the two subtypes of TCGA-HCC patients predicted by the TIDE algorithm. **(K)** Comparison of TIDE scores between the two subtypes of TCGA-HCC patients. **(L)** Unsupervised consistency clustering of the two subtypes of IMvigor210 cohort. **(M)** Comparison of response rates between the two subtypes of IMvigor210 cohort. **(N)** Comparison of survival rates between the two subtypes of the IMvigor210 cohort.

**Figure 3:**
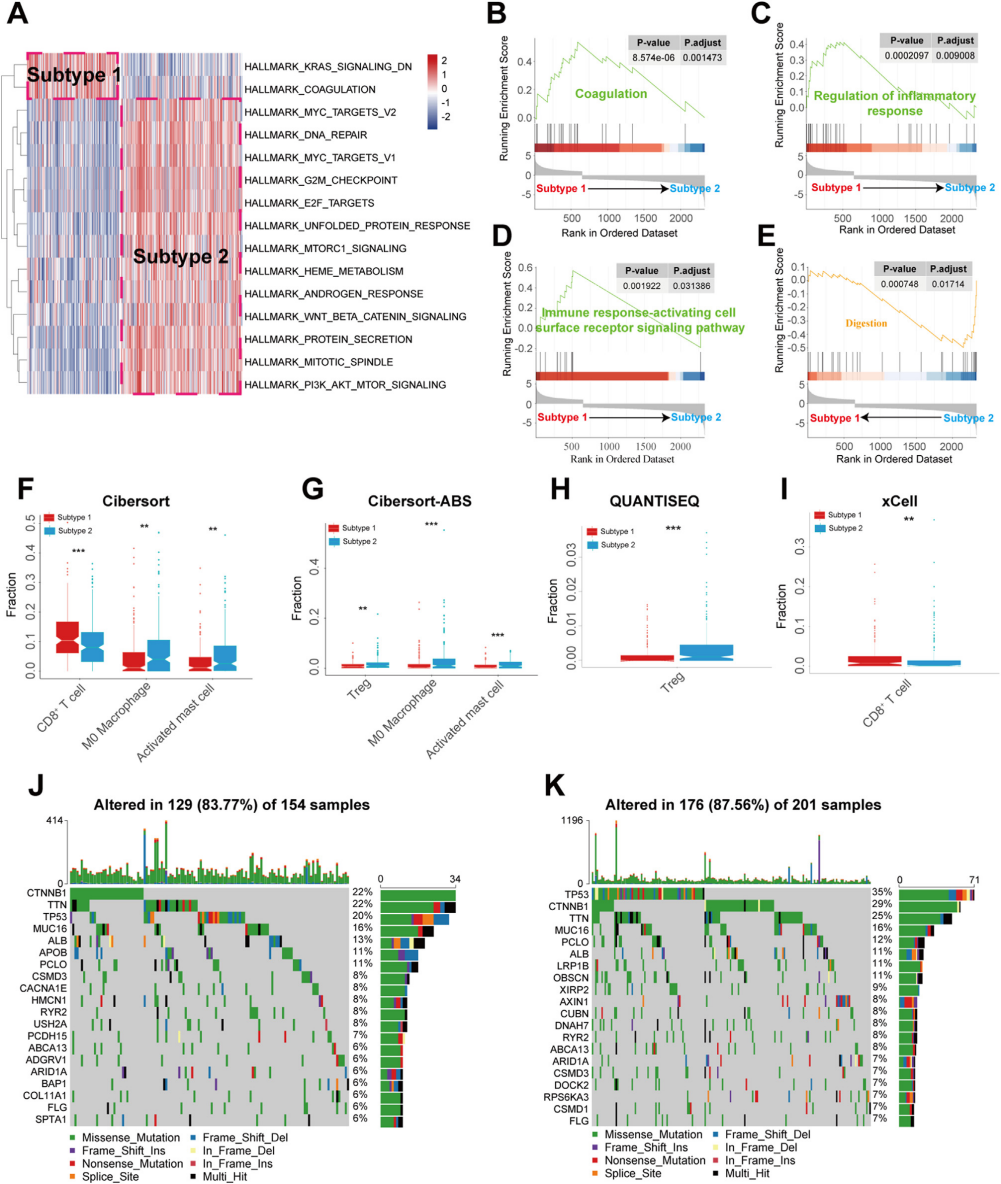
Comparison of ICI-related characteristics of the two subtypes of TCGA-HCC patients. **(A)** Heatmap of the distribution of GSVA scores of the hallmark pathway between the two subtypes. **(B–E)** GSEA analyses of biological processes between the two subtypes. **(F)** Comparison of infiltration levels of CD8^+^ T cells, M0 macrophages, and activated mast cells between the two subtypes under the Cibersort algorithm. **(G)** Comparison of infiltration levels of Tregs, M0 macrophages, and activated mast cells between the two subtypes under the Cibersort-ABS algorithm. **(H)** Comparison of infiltration levels of Tregs between the two subtypes under the QUANTISEQ algorithm. **(I)** Comparison of infiltration levels of CD8^+^ T cells between the two subtypes under the xCell algorithm. **(J)** Waterfall map of the first 20 highly mutated genes in patients with subtype 1. **(K)** Waterfall plot of the first 20 highly mutated genes in patients with subtype 2.

Based on the correlation results of 4872 immune signature scores with TIDE, IPS, MIAS, GEP, IMPRES, xCell-immunescore, and ESTIMATE-immunescore in TCGA-HCC patients, we identified 151 gene sets associated with response to ICIs, including 59 gene sets indicating a good immune response and 92 gene sets indicating a poor immune response (Table S2). Based on the characteristic scores of the 151 immune gene sets, we classified TCGA-HCC patients into subtype 1 and subtype 2 (Fig. 2A). Compared with patients with subtype 2, we found that patients with subtype 1 had more overall survival advantages and higher MIAS, GEP, ESTIMATE-immunescore, IMPRES, IPS, xCell-immunescore scores (Fig. 2B–H). Moreover, we made predictions regarding the response outcome of TCGA-HCC patients using the TIDE score. Patients with TIDE scores above 0 were typically categorized as ICI non-responders, while those with scores below 0 were considered ICI responders (Fig. 2I). The findings indicated that patients exhibiting subtype 1 displayed a higher response rate and lower TIDE score in comparison to those with subtype 2 (Fig. 2J, K). The results are in line with the analysis of the GSE14520 and GSE76427 datasets (Fig. S3, 4). Our method demonstrated greater advantages compared with previously published immunosubtypes^39^^,^^40^ (Tables S3–5). To verify whether the acquired immunosubtypes remain potentially predictive in a real immunotherapy cohort, we applied the same typing method and criteria in the IMvigor210 immunotherapy cohort. These findings indicate that developing tumor subtypes through the analysis of gene sets linked with ICI response has the potential to predict the effectiveness of ICIs (Fig. 2L–N). In terms of pathway analysis, HCC patients with subtype 1 were predominantly associated with “KRAS_SIGNALING_DN”, “COAGULATION”, “regulation of inflammatory response”, and “immune response-activating cell surface receptor signaling pathway” (Fig. 3A–D). In comparison, HCC patients with subtype 2 were associated with “MTORC1_SIGNALING”, “WNT_BETA_CATENIN_SIGNALING”, “PI3K_AKT_MTOR_SIGNALING”, and “digestion” (Fig. 3A–E). When examining tumor-infiltrating immune cells, patients with subtype 1 exhibited a larger proportion of CD8^+^ T cells (Fig. 3F–I). On the other hand, patients with subtype 2 showed higher levels of Tregs, M0 macrophages, and activated mast cells (Fig. 3F–H). In regard to somatic mutations, our analysis of the frequency of the top 20 highly mutated genes revealed that patients with subtype 2 had a higher TP53 mutation rate compared with patients with subtype 1 (Fig. 3J, K).

### Subtype 1-associated natural killer (NK) cells were potentially predictive of ICIs

**Figure 4:**
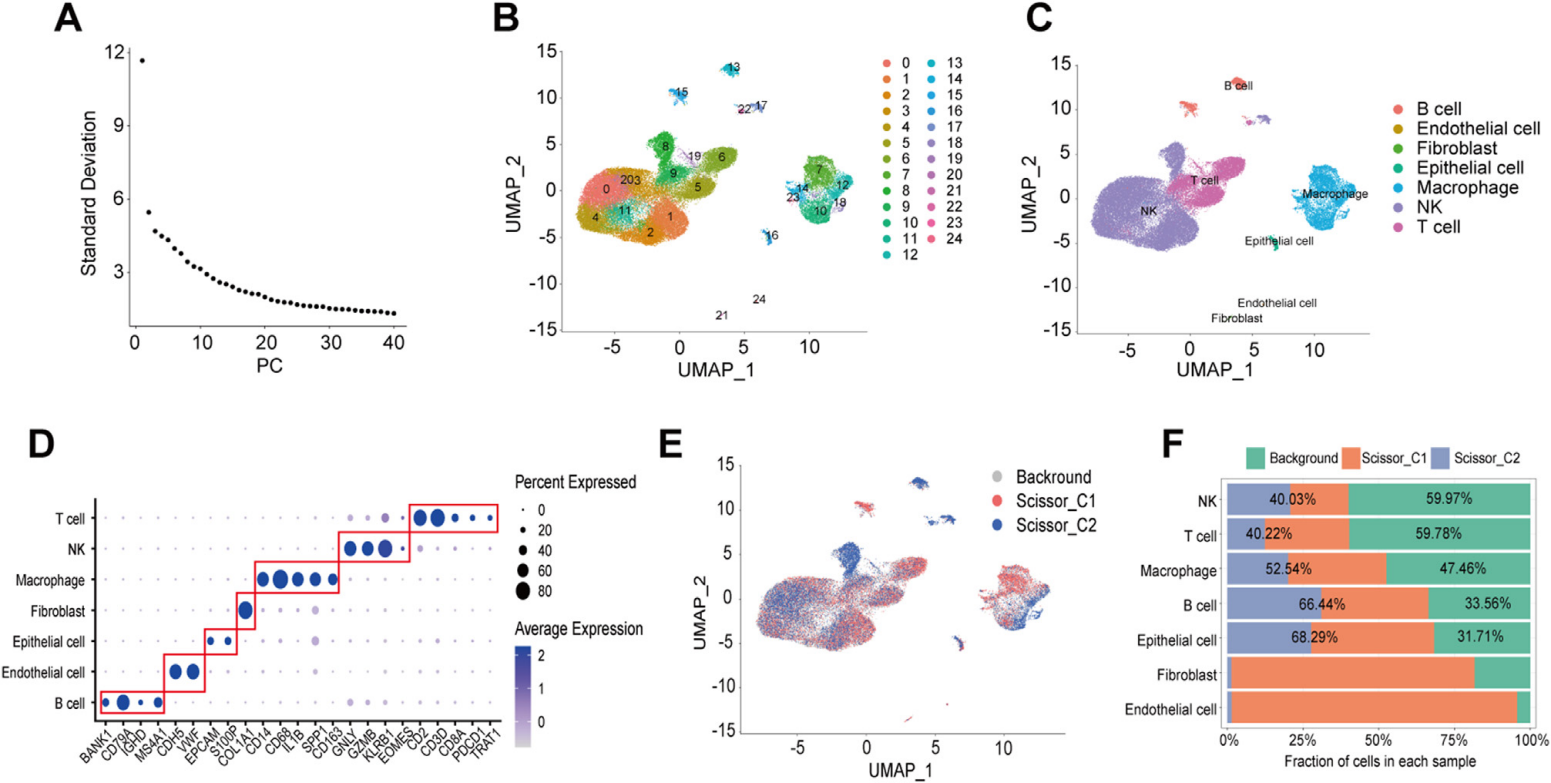
Identification of immune subtype-associated cells. **(A)** Number of principal components versus standard deviation under PCA analysis. **(B)** Visualization of cell clustering under the UMAP algorithm. **(C)** Distribution of annotated cells. **(D)** Average expression of marker genes on each cell. **(E)** Distribution of immune subtype-associated versus non-associated cells. **(F)** Immunosubtype-associated cells in each cell.

**Figure 5:**
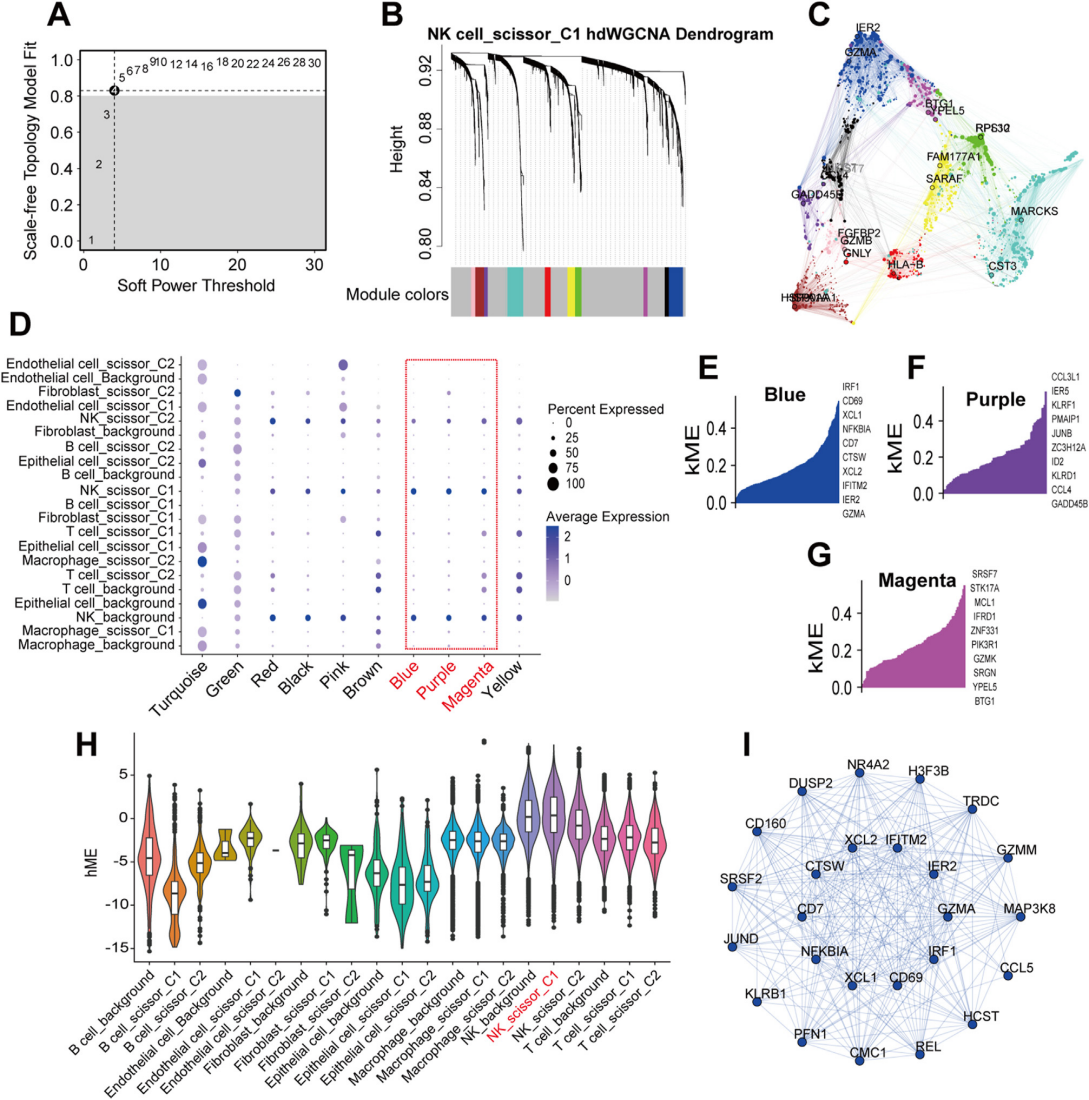
Identification of genes specifically expressed on NK_scissor_C1 cells. **(A)** Variation of scale-free topology fitting with different soft thresholds. **(B)** Clustering of each gene module under hierarchical clustering. **(C)** Distribution of the top 25 hub genes in each gene module. **(D)** Distribution of activation scores of each gene module in each cell. **(E)** The top 10 high connectivity genes in the blue module. **(F)** Top 10 high connectivity genes in the purple module. **(G)** Top 10 high connectivity genes in the magenta module. **(H)** Comparison of activation scores of the blue module in each cell. **(I)** Interaction of the top 25 high connectivity genes in the blue module.

**Figure 6:**
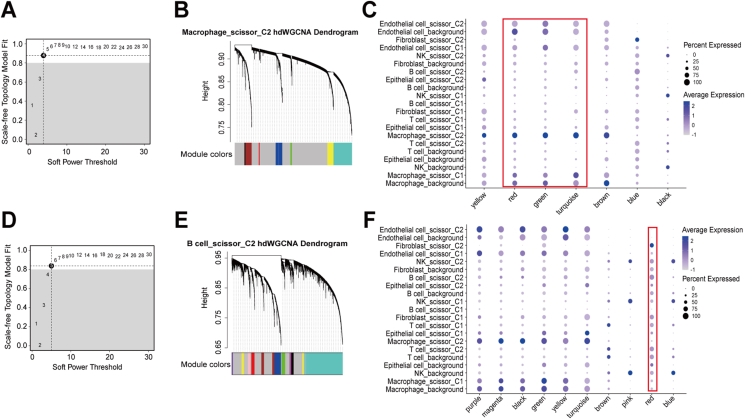
Determination of the cell-specific expression modules of Macrophage_scissor_C2 and B cell_scissor_C2. **(A)** Variation of scale-free topology fitting with different soft thresholds using Macrophage_scissor_C2 as the target cell. **(B)** Clustering of each gene module under hierarchical clustering using Macrophage_scissor_C2 as the target cell. **(C)** Distribution of activation scores of each gene module across cells with Macrophage_scissor_C2 as the target cell. **(D)** Variation of scale-free topology fitting with different soft thresholds with B cell_scissor_C2 as the target cell. **(E)** Clustering of each gene module under hierarchical clustering using B cell_scissor_C2 as the target cell. **(F)** Distribution of activation scores of each gene module across cells with B cell_scissor_C2 as the target cell.

**Figure 7:**
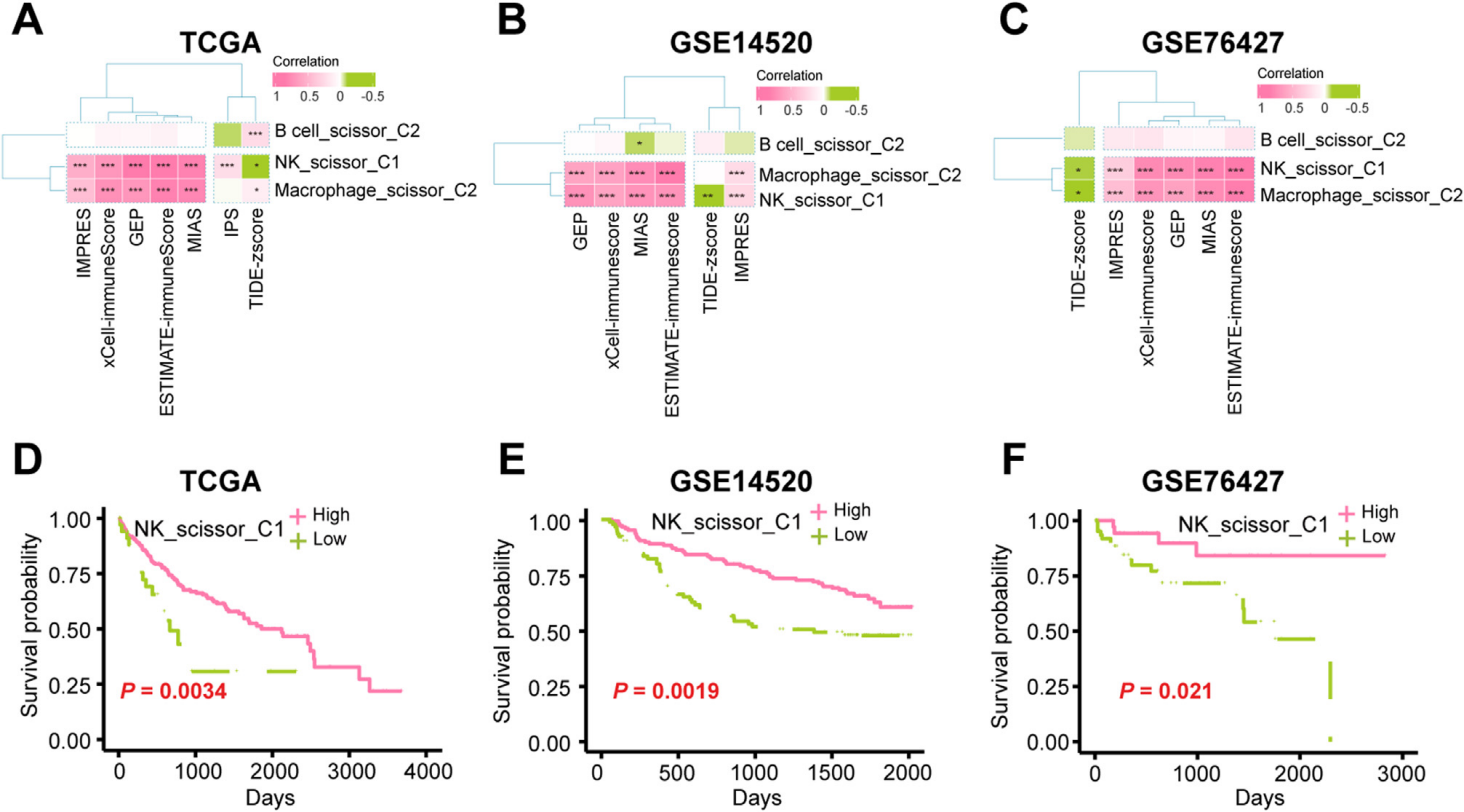
Identification of ICI response-associated cells. **(A)** Correlation of the levels of B cell_scissor_C2, NK_scissor_C1, and Macrophage_scissor_C2 with the seven ICI response-related predictive scores in the TCGA-HCC dataset. **(B)** Correlation of the levels of B cell_scissor_C2, NK_scissor_C1, and Macrophage_scissor_C2 with the six ICI response-related predictive scores in the GSE14520 dataset. **(C)** Correlation of the levels of B cell_scissor_C2, NK_scissor_C1, and Macrophage_scissor_C2 with the six ICI response-related predictive scores in the GSE76427 dataset. **(D)** Comparison of overall survival (OS) of patients in the high and low NK_scissor_C1 level groups in the TCGA-HCC dataset. **(E)** Comparison of OS of patients in the high and low NK_scissor_C1 level groups in the GSE14520 dataset. **(F)** Comparison of OS of patients in the high and low NK_scissor_C1 level groups in the GSE76427 dataset.

After quality control, we obtained 49,489 cells. In addition, we obtained single-cell data after removal of batch effects (Fig. S5). Based on the first 20 principal components obtained by the “Principal Component Analysis (PCA)” algorithm (Fig. 4A), we classified the matrix into 25 subpopulations and annotated them as B cells (13, 15), endothelial cells (24), fibroblasts (21), epithelial cell (16), macrophages (7, 10, 12, 14, 18, 23), NK cells (0, 1, 2, 3, 4, 8, 11, 17, 20), and T cells (5, 6, 9, 19, 22) (Fig. 4B, C). We found that the marker genes of these cells were all specifically expressed in the annotated cells, further indicating the reliability of our annotation results (Fig. 4D and Table S6). After using the CopyKat algorithm, we discovered that 22.36% of the epithelial cells were malignant (Fig. S6). After using the scissor algorithm, we found 21,444 cells associated with immune subtypes, of which the top five cells were NK cells, T cells, macrophages, B cells, and epithelial cells, respectively (Fig. 4E, F). Here, we designated the cells linked with subtype 1 phenotype as scissor_C1, those connected with subtype 2 phenotype as scissor_C2, and cells not related to the immune subtype as background. We conducted hdWGCNA analysis on each of the 14 immune subtype-related cells individually. We discovered that Epithelial cell_scissor_C1, Endothelial cell_scissor_C1, epithelial cell_scissor_C2, Endothelial cell_scissor_C2, and Fibroblast_scissor_C2 cells did not meet the hdWGCNA analysis criteria and were consequently removed. Among the remaining cells related to immune subtype, we did not find any B cell_scissor_C1, Fibroblast_scissor_C1, Macrophage_scissor_C1, T cell_scissor_C1, and NK_scissor_C2 in the specific gene modules (Tables S7–11); hence, their analyses were not the focus of this study. For NK_scissor_C1 cells, an optimal soft threshold of 4 was found (Fig. 5A), resulting in 10 gene modules for analysis (Fig. 5B). The top 25 hub genes within these modules were then visually analyzed for their distribution (Fig. 5C). We observed that only the blue, purple, and magenta modules had a high expression level in NK_scissor_C1 cells, as demonstrated by a bubble plot displaying the distribution of activation scores of the 10 modules across all cells (Fig. 5D). We also identified the genes ranked in the top 10 kME values in these three modules (Fig. 5E–G). To ascertain whether the blue, black, and white modules were significantly expressed in NK_scissor_C1 cells compared with other cells, we generated violin plots. The results showed that only the blue module was expressed specifically at a high level in NK_scissor_C1 cells (Fig. 5H and Table S12). We visualized interactions for the top 25 hub genes in the blue module and found that these genes interacted closely with each other (Fig. 5I and Table S13). Similarly, for Macrophage_scissor_C2 cells, an optimal soft threshold of 4 was found (Fig. 6A), resulting in the identification of seven analyzable gene modules (Fig. 6B). Of these, the red, green, and tortoise modules were found to be specifically activated in Macrophage_scissor_C2 cells (Fig. 6C and Table S14), resulting in the identification of 75 Macrophage_scissor_C2 cell-specific expressed genes across these modules. In relation to B cell_scissor_C2 cells, it was discovered that 10 analyzable gene modules were generated using an optimal soft threshold of 5 (Fig. 6D, E). Of those, only the red module was observed to be exclusively activated in B cell_scissor_C2 cells (Fig. 6F and Table S15). Based on the specifically expressed genes in each cell, we compared the relationship between the levels of NK_scissor_C1 cells, Macrophage_scissor_C2 cells, and B cell_scissor_C2 cells and seven ICI response-related predictive scores, and we found that NK_scissor_C1 cells were positively correlated with immune-promoting scores and negatively correlated with immune-suppressing scores both in the training set (TCGA) (Fig. 7A) and in the test set (GSE14520 and GSE76427) (Fig. 7B, C), and are therefore considered as potential immune cells that can predict response to ICIs. Regarding the remaining two types of immune subtype-associated cells (Macrophage_scissor_C2 and B cell_scissor_C2), their potential to predict ICIs was inconclusive due to conflicting correlations (Fig. 7A–C). Furthermore, the results indicated that elevated levels of NK_scissor_C1 cells correlated with improved overall survival (Fig. 7D–F).

### Docetaxel and thalidomide as potential therapeutic agents targeting NK_scissor_C1 cells

**Figure 8:**
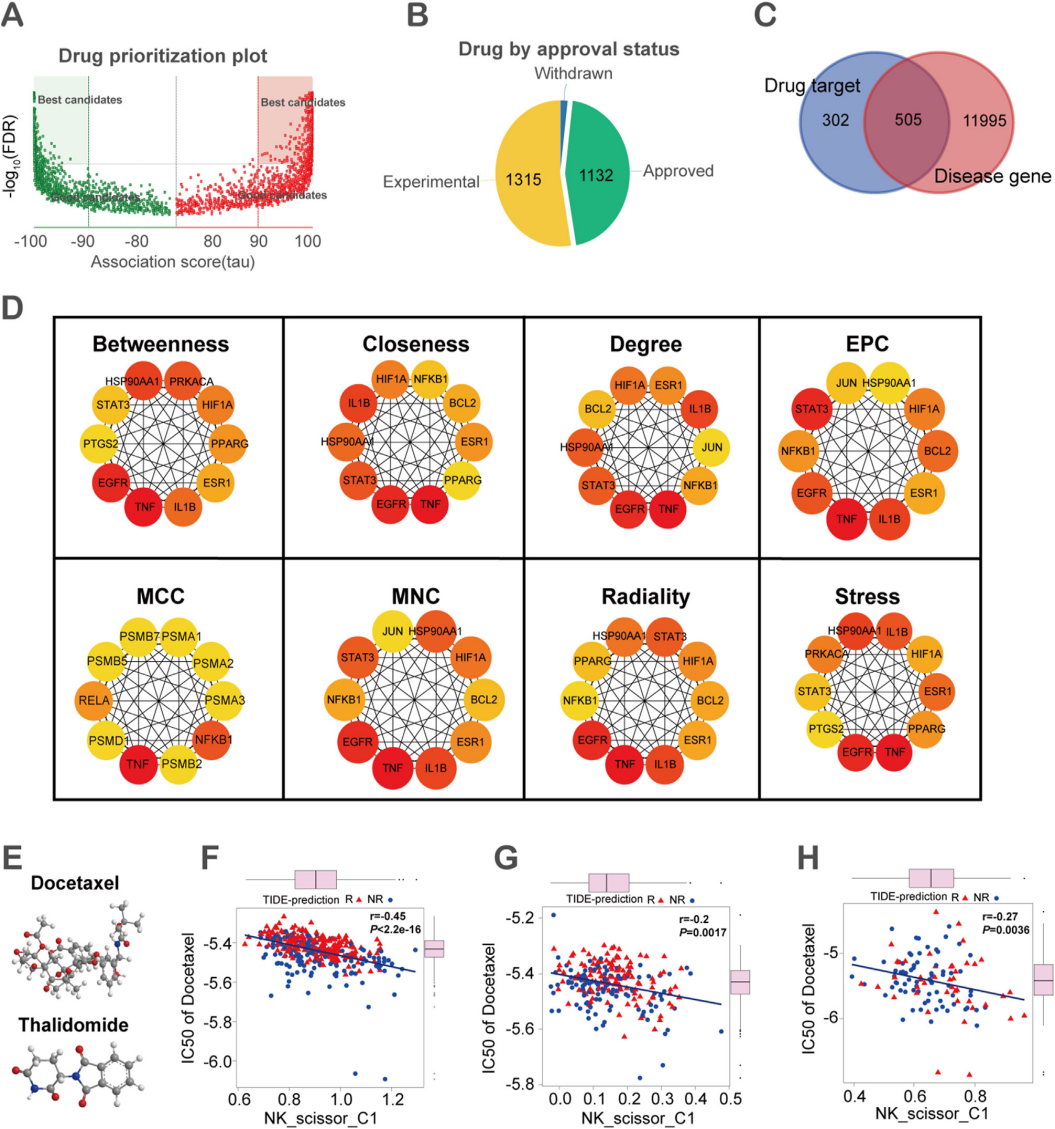
Identification of NK_scissor_C1 cell-associated drug candidates. **(A)** Volcano plot of NK_scissor_C1-associated drug priorities. **(B)** NK_scissor_C1-associated drug usage status. **(C)** The Veen diagram showing intersections of NK_scissor_C1-associated drug targets with HCC-associated genes. **(D)** Interaction network of top 10 significant nodes under 8 predictive node importance algorithms. EPC, edge percolated component; MCC, maximal clique centrality; MNC, maximum neighborhood component. **(E)** 3D structures of the two drug candidates docetaxel and thalidomide. **(F)** Correlation of NK_scissor_C1 levels with drug sensitivity to docetaxel in the TCGA-HCC dataset. **(G)** Correlation of NK_scissor_C1 levels with drug sensitivity to docetaxel in the GSE14520 dataset. **(H)** Correlation of NK_scissor_C1 levels with drug sensitivity to docetaxel in the GSE76427 dataset.

Using the DREIMT database, we obtained 2494 potential drugs that may be involved in the regulation of NK_scissor_C1 cells through 25 genes specifically expressed in NK_scissor_C1 cells, including 1132 approved drugs, 1315 drugs in ongoing trials, and 47 drugs that have been withdrawn (Fig. 8A, B). We extracted 505 candidate genes from 807 drug targets of 1132 approved drugs and 12,500 disease genes associated with HCC for protein interaction analysis (Fig. 8C and Table S16). Based on the results of eight algorithms for predicting the importance of protein nodes, the top 10 genes with the highest number of occurrences were TNF, EGFR, ESR1, HIF1A, HSP90AA1, IL1B, STAT3, NFKB1, BCL2, and PPARG, which in turn led to our identification of 80 drug candidates targeting these genes (Fig. 8D and Table S17). To ensure that these drug candidates have anti-tumor and immunomodulatory effects, we further screened the 80 drug candidates through the DGIdb database and finally retained four drugs, namely thalidomide, lenalidomide, docetaxel, and paclitaxel. Finally, we queried whether these four drugs were relevant to HCC treatment and the current research status through the drugbank database. We found that all four drugs were relevant to HCC treatment, but one study found that lenalidomide was in discontinued status because it was ineffective in the treatment of HCC, and the paclitaxel was withdrawn. Therefore, in response to the above analyses, we identified docetaxel and thalidomide as potential therapeutic agents for targeting NK_scissor_C1 cells and mapped their 3D structures (Fig. 8E). To investigate whether the sensitivity of HCC patients to these two drugs affects the level of NK_scissor_C1 cells, we analyzed the drugs built into the “pRRophetic” package accordingly but found that only docetaxel was included, as thalidomide was absent from the list of drugs. We found that the IC50 value of docetaxel was negatively correlated with the NK_scissor_C1 cell level (Fig. 8F–H), indicating that the NK_scissor_C1 cell level of HCC patients increases with the sensitivity of docetaxel.

## Discussion

Although the idea of classification has become commonplace in omics studies, most of these studies focus on the choice of classification methods, while few studies will consider the choice of classification features with a purpose. In our study, we first used seven predictive scores related to response to ICIs as a tool to measure whether the immune gene set can predict response to ICIs in HCC patients, and the results confirmed that our immune signature scores are able to classify HCC patients well into two immune subtypes with differences in immune response. In addition, we found significant differences in certain pathways, tumor-infiltrating immune cells, and gene mutations between the two subtypes. Mutations in the KRAS gene can trigger the development of invasive HCC,^41^ and up-regulation of the KRAS pathway has been reported in patients with tumors that do not respond to PD-1 blocker therapy.^42^ He et al found that coagulation-related features can predict the response of HCC patients to ICIs.^43^ mTORC1 regulates protein synthesis, glucose and lipid metabolism, and autophagy, and has been shown to influence the development of HCC.^44^ Wnt/β-catenin signaling has been considered a target pathway for the treatment of HCC because its activation promotes the proliferation, migration, and invasion of HCC cells.^45^ A study on metabolic network classification in HCC discovered subtypes linked to PI3K/AKT/mTOR signaling activation exhibited the least favorable survival rates.^46^ Among several subtypes of macrophage, M0 macrophage plays a role in the poorer prognosis of HCC patients and could be a potential target for immunotherapy.^47^^,^^48^ Mast cells typically emerge during liver injury and are stimulated to secrete proteases and mediators that exacerbate liver disease and lead to a poorer prognosis.^49^^,^^50^ The appearance of Tregs is often associated with lenvatinib, as the immunomodulation of the tumor infiltration by lenvatinib is associated with a decrease in Tregs.^51^^,^^52^ Genetic mutations not only affect the prognosis of tumor patients but also influence immune responses. Mutations in the TP53 gene were found to be associated with poorer prognosis in HCC subtypes, and more importantly, higher mutation rates in TP53 create an immunosuppressive microenvironment.^53^^,^^54^ The results showed that patients with subtype 1 exhibited immune-promoting signals and immune cells. On the other hand, patients with subtype 2 displayed increased active scores of cancer-linked pathways and heightened levels of immunosuppressive cells. This explains why subtype 1 patients have more immune-promoting features, while subtype 2 patients exhibit poorer overall survival and low immune levels. More importantly, these findings offer further support for the accuracy of the immunophenotyping results.

It is widely acknowledged that cellular heterogeneity affects HCC immunotherapy, which was likewise illustrated in this study. Although conventional bulk sequencing methods have found differences in patients with responding ICIs based on their immune subtypes, it remains unclear whether these differences target all immune cells at the cellular level. Indeed, our findings reveal that only NK cells that are linked to hyperimmune subtypes may be potentially indicative of ICIs within the numerous cells. The quantification of such cells was based on 25 key highly interacting immune-related genes, most of which have immune-promoting effects within them. For example, an excess of the chemokine CCL5 increases antigen-specific CD8^+^ T cells, which exert anti-tumor immunity.^55^ Defects in CD160 have been implicated as a potential cause of tumor immune escape, suggesting its therapeutic potential in inhibiting tumor metastasis.^56^ IRF-1 increases the migration of CD8^+^ T cells, NK cells, and NKT cells, and activates the secretion of IFN-γ by NK and NKT cells, thereby inducing tumor apoptosis.^57^

Most chemotherapeutic agents have an immunostimulatory effect, which inhibits or activates the anti-tumor effect of immune cells.^58^ Among them, it has been found that low-dose docetaxel promotes cytotoxic lymphocyte infiltration, inhibits myeloid-derived suppressor cells, and effectively polarizes myeloid-derived suppressor cells to the M1 phenotype, which further enhances anti-tumor immune efficacy.^59^ In Chen's study, it was also found that docetaxel could induce the production of M1-type macrophages to achieve effective reversal of immunosuppression.^60^ Although ICIs are a breakthrough in the treatment of HCC in recent years, some limitations need to be overcome and resolved by combination therapies.^61^ Docetaxel has been reported to promote T-cell infiltration in a cGAS/STING-dependent manner, which improves the clinical efficacy of immunotherapy.^62^

Overall, our study offers several advantages. Firstly, we classified HCC patients into different immune subtypes using immune gene set signature scores as classification features and ICI response-related predictive scores as a reference. This process enables us to determine the characteristics of the subtypes by targeting the attributes of the classification features. We concentrated on classifying attributes more than enhancing the classification effect by improving the classification method. Indeed, we have also tentatively demonstrated that the immunological subtypes we obtained do have the potential to predict ICI response. Secondly, the consideration of cellular heterogeneity is seldom reflected in many typing studies. Our study used single-cell sequencing to integrate immune subtypes with cellular categories. Since single-cell sequencing sparsity and noise could lead to inaccurate gene-to-gene correlations, we used the hdWGCNA algorithm to greatly reduce sparsity and preserve cellular heterogeneity. This approach led to the identification of cell-specific hub genes at the cellular level. Finally, we used multiple databases trained and validated against each other to obtain the final potential therapeutic agents while obtaining ICI response-associated candidate cells.

However, it is important to note that our study has limitations. Firstly, our finding that the level of the subtype 1-associated NK cell subset is positively correlated with docetaxel sensitivity and that this NK cell subset promotes response to ICIs is correlational. As with most bioinformatics studies, it is not possible to determine a causal relationship currently. Therefore, the existence of a causal relationship cannot be determined based on our findings. The study's findings were solely based on bioinformatics analysis and have not yet been validated through animal experiments or clinical trials. To address these limitations, future tasks should include exploring mechanistic studies to investigate the interaction mechanism between docetaxel and subtype 1-associated NK cell subsets. This involves understanding the impact of docetaxel on the levels of NK cell subsets and their contribution to treatment response to ICIs. Subsequently, clinical studies are conducted to assess the effectiveness and safety of docetaxel in combination with ICIs for tumor patients. These studies may comprise randomized controlled trials and preclinical studies to determine the optimal treatment regimen and patient selection criteria.

In conclusion, our study has revealed the cell types that potentially affect ICIs and identified potential drugs by combining bulk sequencing and single-cell sequencing. This finding will provide a scientific reference for future studies of ICIs in HCC treatment.

## CRediT authorship contribution statement

G.C.L. and A.L.H. designed the study. G.C.L. and B.X. performed the analysis. G.C.L. and W.W. wrote the manuscript. X.N.Z., H.L., and J.L.D. performed the validation in the independent cohort. C.Z., K.J.L., Y.Z., T.Y.Z., A.B.L., Y.L., and J.F. prepared the figures and tables. G.C.L., B.X., W.W., and A.L.H. revised the manuscript. All authors reviewed the manuscript and approved the final version.

## Conflict of interests

Ailong Huang is editor-in-chief for *Genes & Diseases*, and he was not involved in the editorial review or the decision to the manuscript. The other authors declared no known financial interests or personal relationships of the authors that may influence the work described in this article.

## Funding

The study was financially supported by the Science and Technology Research Programme Project of Chongqing Municipal Education Commission of China (No. KJQN202300423), the National Youth Science Foundation Project (China) (No. 82204159), the Science and Technology Project of Sichuan Provincial Administration of Traditional Chinese Medicine (China) (No. 2023MS047), the Program for Youth Innovation in Future Medicine, Chongqing Medical University (No. W0150), and the Intelligent Medicine Research Project of Chongqing Medical University (No. ZHYX202223).

## Data accessibility

All of the data being studied can be accessed through these tools, including TCGA (http://xenabrowser.net/), GSE14520 (https://www.ncbi.nlm.nih.gov/geo/query/acc.cgi?acc=GSE14520), GSE76427 (https://www.ncbi.nlm.nih.gov/geo/query/acc.cgi?acc=GSE76427), GSE166635 (https://www.ncbi.nlm.nih.gov/geo/query/acc.cgi?acc=GSE166635), GSE162616 (https://www.ncbi.nlm.nih.gov/geo/query/acc.cgi?acc=GSE162616), MSigDB (http://gsea-msigdb.org/gsea/index.jsp), CellMarker2.0 (http://117.50.127.228/CellMarker/), DRElMT (http://www.dreimt.org/query/drug-prioritization), GeneCards (http://genecards.org/), String (http://cn.string-db.org/cgi/input?sessionId=bq3ONpHVnJ16&input_page_show_search=on)、DrugBank (http://go.drugbank.com/), DGIdb (http://dgidb.org/), and Pubchem (http://pubchem.ncbi.nlm.nih.gov/).
