# Supplementary material for: Comprehensive analysis of immune subtype characterization on identification of potential cells and drugs to predict response to immune checkpoint inhibitors for hepatocellular carcinoma
Source: Genes Dis. 2024 Nov 27;12(3):101471. doi: 10.1016/j.gendis.2024.101471 (PMC11907441; doi:10.1016/j.gendis.2024.101471)
Supplement: Multimedia component 1 [file mmc1.docx]

**Figure S1** Schematic diagram of Scissor algorithm.

**Figure S2** Schematic diagram of hdWGCNA algorithm.

**Figure S3** Validation of immunosubtyping in GSE14520.

**Figure S4** Validation of immunosubtyping in GSE76427.

**Figure S5** UMAP visualization of cell clustering before and after batch correction.

**Figure S6** CopyKat algorithm identifies normal and tumor cells in epithelial cells.

**Table S1** HCC-related genes obtained from GeneCards database.

**Table S2** 151 ICI response-related gene sets.

**Table S3** Comparison of our study with similar previous studies in the TCGA dataset.

**Table S4** Comparison of our study with similar previous studies in the GSE14520 dataset.

**Table S5** Comparison of our study with similar previous studies in the GSE76427 dataset.

**Table S6** List of marker genes for cellular annotation.

**Table S7** The information about each gene module associated with B cell_scissor_C1 cells.

**Table S8** The information about each gene module associated with fibroblast_scissor_C1 cells.

**Table S9** The information about each gene module associated with macrophage_scissor_C1 cells.

**Table S10** The information about each gene module associated with T cell_scissor_C1 cells.

**Table S11** The information about each gene module associated with NK_scissor_C2 cells.

**Table S12** The information about each gene module associated with NK_scissor_C1 cells.

**Table S13** 25 hub genes in the blue module of NK_scissor_C1 cells.

**Table S14** The information about each gene module associated with macrophage_scissor_C2 cells.

**Table S15** The information about each gene module associated with B cell_scissor_C2 cells.

**Table S16** 505 genes at the intersection of drugs and disease.

**Table S17** The drug candidates targeting the top 10 highly connected genes.
