# Supplementary material for: Comprehensive analysis of immune subtype characterization on identification of potential cells and drugs to predict response to immune checkpoint inhibitors for hepatocellular carcinoma
Source: Genes Dis. 2024 Nov 27;12(3):101471. doi: 10.1016/j.gendis.2024.101471 (PMC11907441; doi:10.1016/j.gendis.2024.101471)
Supplement: Multimedia component 2 [file mmc2.docx]

**Scissor algorithm**: This algorithm utilizes the principle of network regularization sparse regression model to identify phenotypically related cells (**Fig. S1**). The process is as follows:


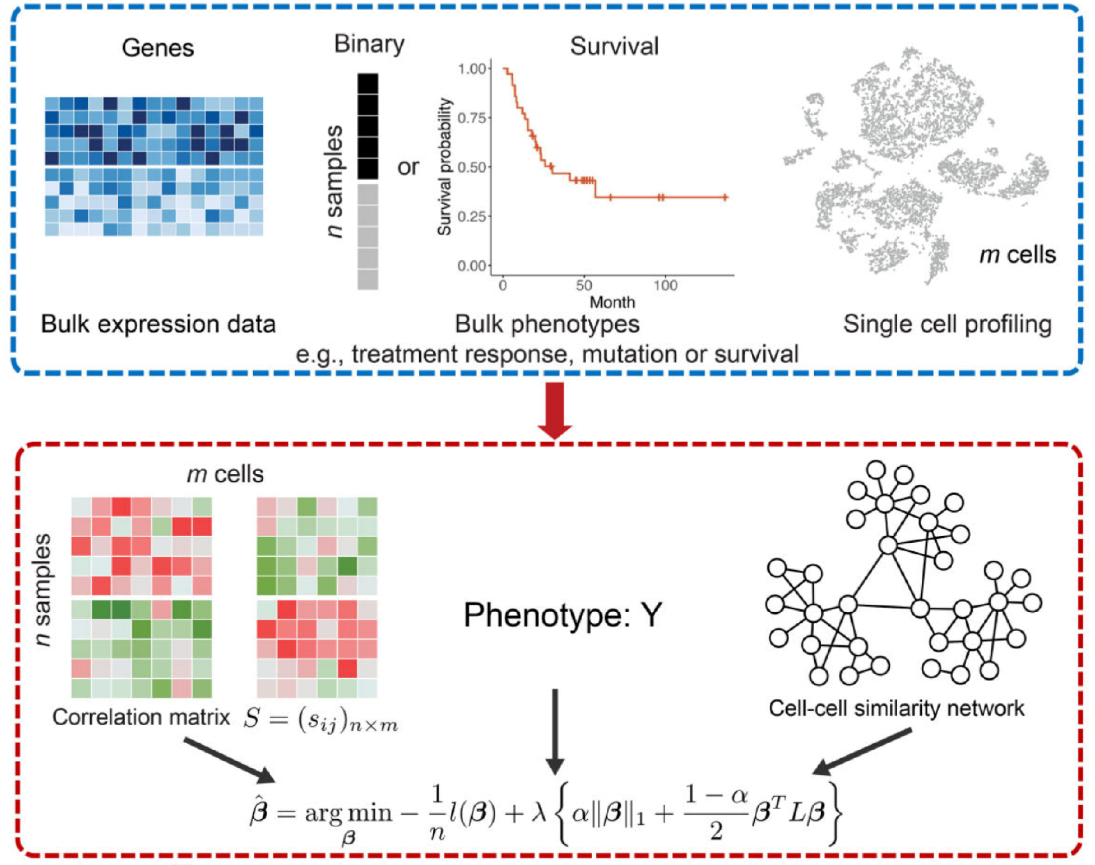


**Figure S1** Schematic diagram of Scissor algorithm.

1. **Input data:** bulk sequencing data from TCGA cohort (transcriptome data, immunosubtype status), single-cell sequencing data from HCC patients.
2. **Determination of immunosubtype-associated cells：** $n$ denote the number of samples from bulk sequencing and $m$ denote the number of cells of single-cell sequencing, a Pearson analysis was performed on the single-cell sequencing matrix versus the bulk sequencing matrix based on common genes, thus obtaining the correlation $S_{ij}$ between samples $i$ and cells $j$. where $\left( s_{ij} \right)_{n\times m}$ is the correlation matrix between the bulk sequencing matrix and the single cell sequencing matrix, denoted by $S$. Use "Seurat" package built-in shared nearest-neighbor graph function to draw the cell-to-cell similarity network G, get the adjacency matrix $\left( a_{ij} \right)_{m\times m}$, denoted by $A$, and the connectivity of the cell similarity network $\left( d_{ij} \right)_{m\times m}$, denoted by $D$, based on the adjacency matrix and connectivity, the symmetric normalized Laplace matrix is obtained (**Equation 1-1**). In this study, the log-likelihood function of logistic regression is taken to obtain (**Equation 1-2**). Finally, the optimization model of Scissor's algorithm is obtained by substituting Eqs. 1-1 and 1-2 into Eq. 1-3 to solve for non-zero coefficients. In this study, cells with $\beta$>0 were defined as subtype 1-associated cells, while cells with $\beta$<0 were defined as subtype 2-associated cells.

$L=D^{-\frac{1}{2}}(D-A)D^{-\frac{1}{2}}=I-D^{-\frac{1}{2}}AD^{-\frac{1}{2}}$ (1-1)

$l(\beta)=\sum_{i=1}^{n} \left[ y_{i}\beta^{T}S_{i}-\log\left( 1+\exp\left( \beta^{T}S_{i} \right) \right] \right.$($y_{i}$ is the true attribute of sample $i$, denoted by 0 or 1; $S_{i}$ is the correlation coefficient between the sample $i$ and the m cells, and $\beta^{T}S_{i}$ is the predicted value of the model for sample $i$) (1-2)

$\hat{\beta}=\underset{\beta}{\arg min}-\frac{1}{n}l(\beta)+\lambda\left\{ \alpha\parallel\beta\parallel_{1}+\frac{1-\alpha}{2}\beta^{T}L\beta\right\}$ ($\lambda$ is a regularization parameter used to balance the trade-off between fitting the data and penalizing the model complexity; $\alpha$ is a parameter controlling the weight between the 1-regularization term and the network regularization term; $\parallel\cdot\parallel_{1}$denotes the 1-paradigm number, which is used to sparsify the parameter $\lambda$) (1-3)
