# Supplementary material for: Comprehensive analysis of immune subtype characterization on identification of potential cells and drugs to predict response to immune checkpoint inhibitors for hepatocellular carcinoma
Source: Genes Dis. 2024 Nov 27;12(3):101471. doi: 10.1016/j.gendis.2024.101471 (PMC11907441; doi:10.1016/j.gendis.2024.101471)
Supplement: Multimedia component 3 [file mmc3.docx]

**hdWGCNA algorithm:** analyze high-dimensional transcriptomic data such as single-cell and spatial RNA-seq. hdWGCNA provides functions for network inference, gene module identification, gene enrichment analysis, statistical tests, and data visualization. In addition to conventional single-cell RNA-seq, hdWGCNA is able to perform isoform-level using long-read single-cell data network analysis (**Fig. S2**).


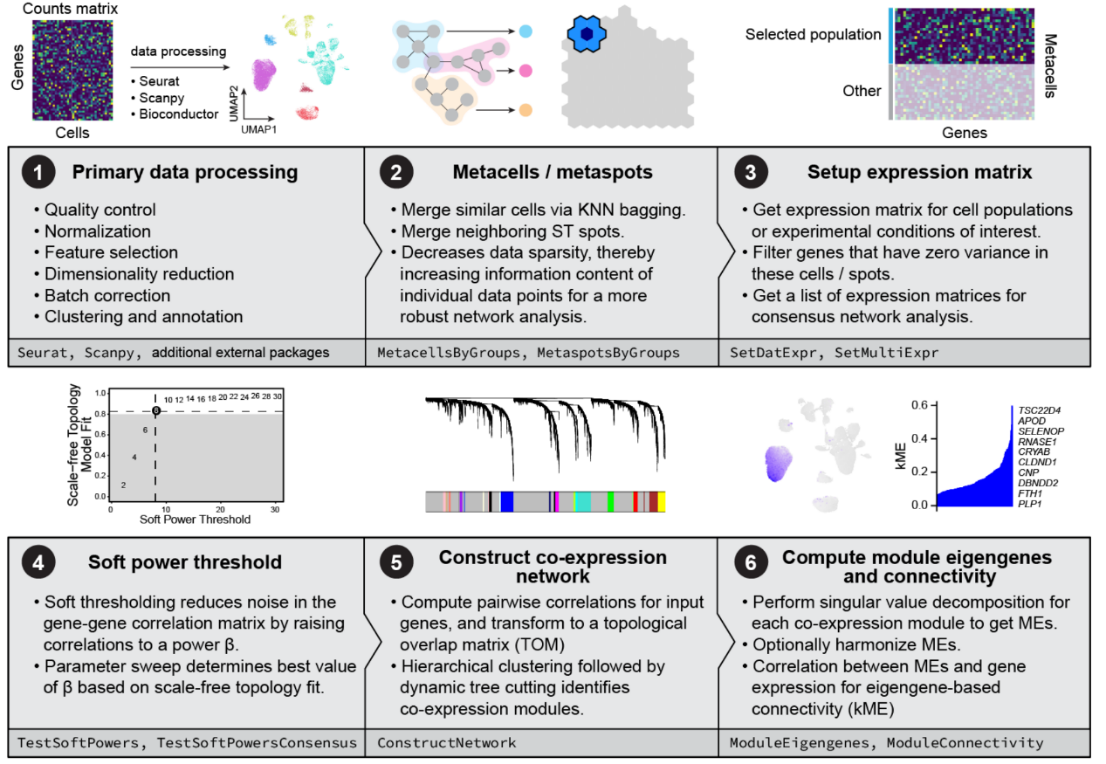


**Figure S2** Schematic diagram of hdWGCNA algorithm.

**The specific processes include:**

1. **Forming metacells:** single-cell transcriptomes are bootstrap polymerized to form metacells. Single-cell gene expression datasets usually contain more zero-valued entries than non-zero values, implying that these datasets are sparse. The sparsity of a gene expression matrix $\text{sparsity}$ is defined in **Equation 1-1**. Given a non-normalized count matrix with genes $N_{g}$ and cells $N_{c}$, the sparsity is the sum of all zero-valued elements

$\text{sparsity }=\frac{\sum_{i=1}^{N_{g}} \sum_{j=1}^{N_{c}} \left\{ \begin{aligned} &1 &&\text{ if }X_{i,j}=0 \\ &0 &&\text{ else } \end{aligned} \right.}{N_{g}\times N_{c}}$($N_{g}$ denotes number of genes；$N_{c}$ denotes number of cells.) (1-1)

1. **Calculate Co-Expression Networks:** hdWGCNA constructs co-expression networks and identifies gene modules based on the weighted gene co-expression network analysis. The gene-gene adjacency matrix A is obtained by calculating the pairwise correlations of genes in G in the metacellular expression matrix M. Consider an arbitrary gene pair ($i$*,*$j$)∈ G. Symbolic correlations can be represented by $a_{i,j}$ (**Equation 1-2**). To emphasize the gene-to-gene correlations, a soft threshold $\beta$ is introduced and $\tilde{\alpha}_{i,j}$ was used to preserve the correlation symbols of these genes (**Equation 1-3**). The final co-expression network is computed as a signed TOM (**Equation 1-4**). The TOM describes the shared neighbors between a pair of genes ($i$,$j$).

$a_{i,j}=\frac{1+\mathrm{cor}\left( x_{i},x_{j} \right)}{2}$ (1-2)

$\begin{aligned} \alpha_{i,j}=\left( a_{i,j} \right)^{\beta} \\ \tilde{\alpha}_{i,j}=\alpha_{i,j}\times\mathrm{sign}\left( \mathrm{cor} \left( x_{i},x_{j} \right) \right) \end{aligned}$ (1-3)

$\mathrm{TOM}_{i,j}^{\text{signed }}=\frac{\left| \alpha_{i,j}+\sum_{u\neq i,j} \tilde{\alpha}_{i,u}\tilde{\alpha}_{u,j} \right|}{min\left( k_{i},k_{j} \right)+1-\left| \alpha_{i,j} \right|}$ (1-4)

1. **Calculation of Module Eigengenes (MEs):** MEs can be used to reflect the gene expression of a given co-expressed module, thus obtaining information about the activity of each module in each cell. The MEs of a module can be solved by singular value decomposition, which is equal to the first vector of the right singular vector.
