# Supplementary material for: Comprehensive analysis of immune subtype characterization on identification of potential cells and drugs to predict response to immune checkpoint inhibitors for hepatocellular carcinoma
Source: Genes Dis. 2024 Nov 27;12(3):101471. doi: 10.1016/j.gendis.2024.101471 (PMC11907441; doi:10.1016/j.gendis.2024.101471)
Supplement: Multimedia component 4 [file mmc4.docx]

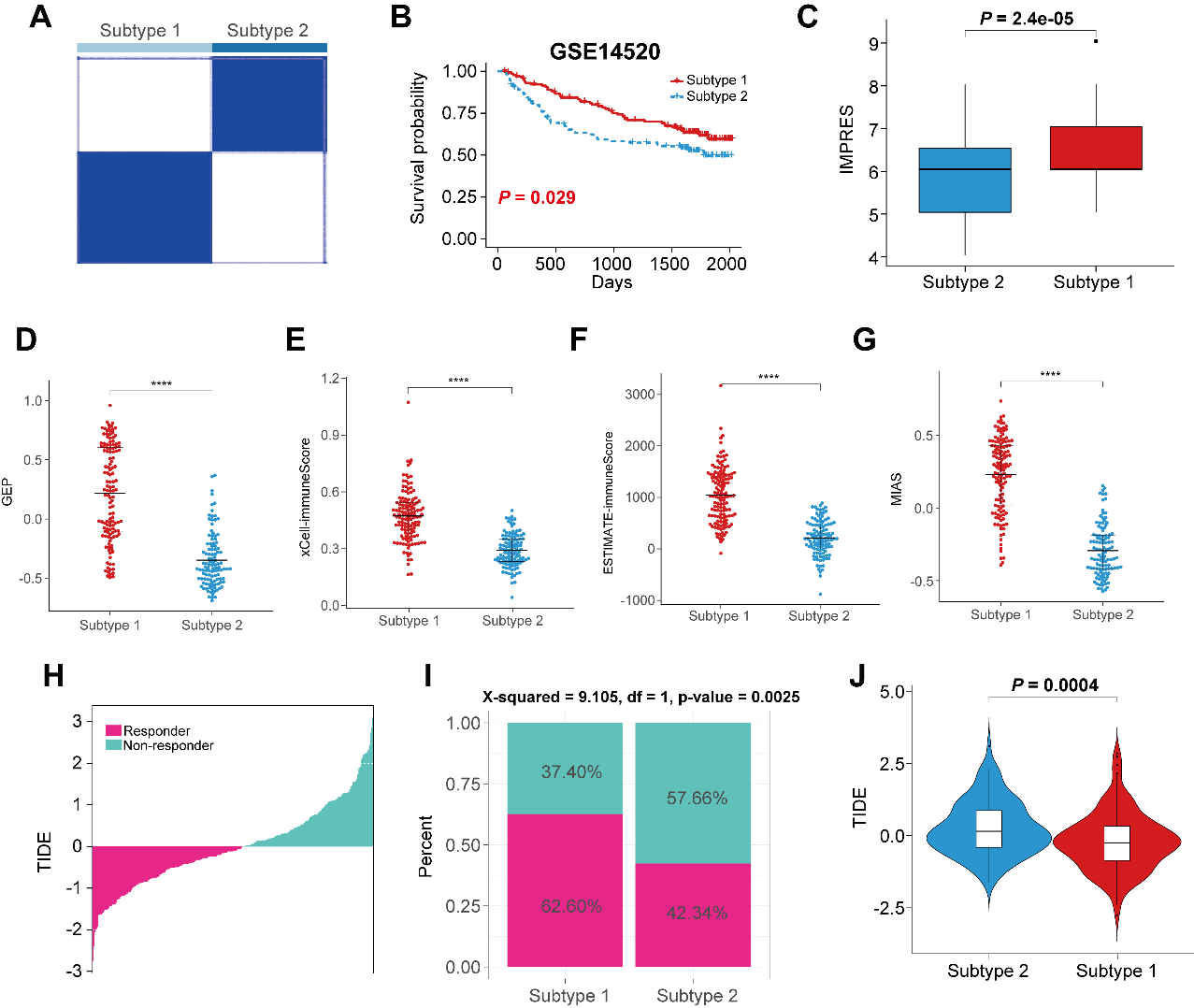


**Figure S3** Validation of immune subtypes based on ICIs response-related gene set feature scores in GSE14520. (**A**) Unsupervised consistent clustering of the two subtypes. (**B**) Comparison of OS between the two subtypes. (**C**) Comparison of IMPRES scores between the two subtypes. (**D**) Comparison of GEP scores between the two subtypes. (**E**) Comparison of xCell-immuneScore scores between the two subtypes. (**F**) Comparison of ESTIMATE-immuneScore scores between the two subtypes. (**G**) Comparison of MIAS scores between the two subtypes. (**H**) Distribution of TIDE scores between responders and non-responders. (**I**) Comparison of response rates between the two subtypes. (**J**) Comparison of TIDE scores between the two subtypes.
