# Supplementary material for: Comprehensive analysis of immune subtype characterization on identification of potential cells and drugs to predict response to immune checkpoint inhibitors for hepatocellular carcinoma
Source: Genes Dis. 2024 Nov 27;12(3):101471. doi: 10.1016/j.gendis.2024.101471 (PMC11907441; doi:10.1016/j.gendis.2024.101471)
Supplement: Multimedia component 6 [file mmc6.docx]

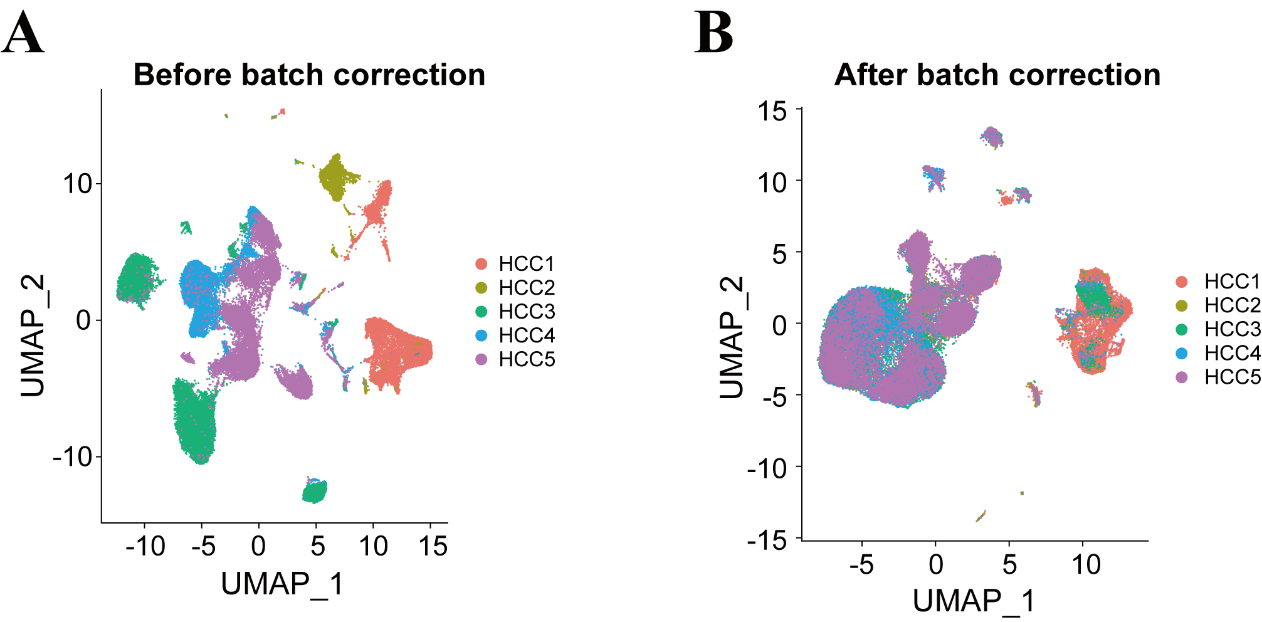


**Figure S5** UMAP visualization of cell clustering before and after Harmony algorithm. (A) UMAP visualization of cells before batch correction. (B) UMAP visualization of cells after batch correction.
