# Supplementary figures and images for: Comprehensive analysis of immune subtype characterization on identification of potential cells and drugs to predict response to immune checkpoint inhibitors for hepatocellular carcinoma
Source: Genes Dis. 2024 Nov 27;12(3):101471. doi: 10.1016/j.gendis.2024.101471 (PMC11907441; doi:10.1016/j.gendis.2024.101471)

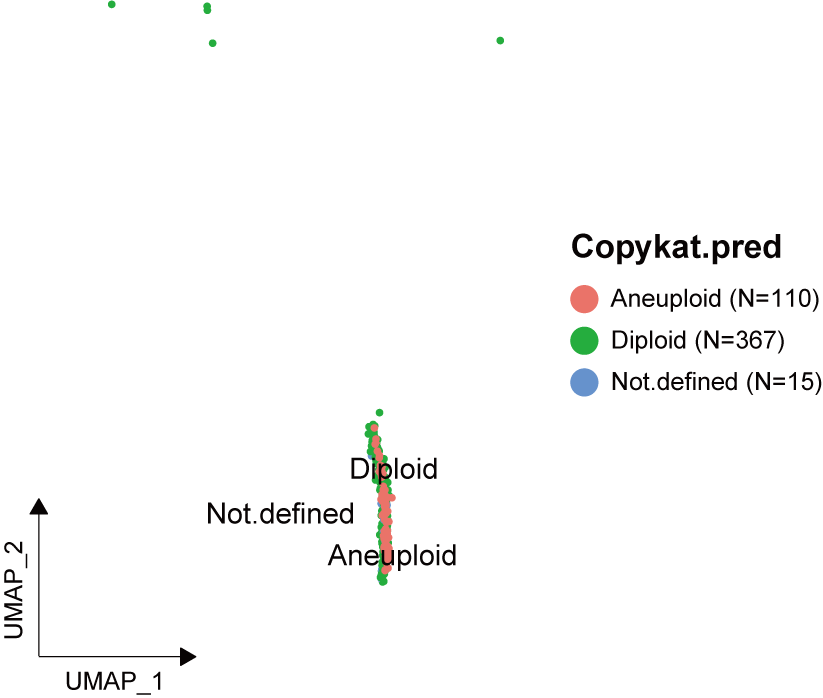


**Figure S6** CopyKat algorithm identifies normal and tumor cells in epithelial cells.

Supplement: Multimedia component 7 [file mmc7.docx]
